# Hyperoncotic Albumin (20%) Versus Crystalloids for Mortality in Adults with Sepsis and Septic Shock: An Updated Systematic Review and Meta-Analysis

**DOI:** 10.3390/medsci14050550

**Published:** 2026-09-07

**Authors:** Christian J. Wiedermann, Gianni Turcato, Arian Zaboli, Michael Joannidis

**Affiliations:** 1 Institute of General Practice and Public Health, Claudiana—College of Health Professions, 39100 Bolzano, Italy; 2 Intermediate Care Unit, Department of Internal Medicine, Hospital Alto Vicentino (AULSS-7), 36014 San-torso, Italy; 3 Health Professions Management, South Tyrolean Health Authority (SABES-ASDAA), 39100 Bolzano, Italy; 4 Division of Intensive Care and Emergency Medicine, Department of Internal Medicine, Medical University of Innsbruck, 6020 Innsbruck, Austria

**Keywords:** human albumin, hyperoncotic albumin, septic shock, sepsis, fluid resuscitation, crystalloids, mortality, meta-analysis

## Abstract

**Background/Objective:** Hyperoncotic albumin (20%) has been proposed for sepsis; however, the randomized evidence is inconclusive, and the 2026 Surviving Sepsis Campaign guidelines now suggest crystalloids alone while permitting albumin on a case-by-case basis. We updated the evidence, restricting the intervention to hyperoncotic albumin (≥20%) and the comparator exclusively to crystalloids, and examined whether the benefit was confined to septic shock. **Methods:** We searched PubMed, EMBASE, and Cochrane CENTRAL (March 2024–March 2026), supplementing a previously published meta-analysis. Randomized trials of hyperoncotic albumin versus crystalloids in adults with sepsis or septic shock reporting all-cause mortality were included. The primary outcome was mortality at the longest follow-up, pooled as risk ratios (RRs) using random-effects models. Given negligible heterogeneity, the estimate was reported under standard, Hartung–Knapp–Sidik–Jonkman (HKSJ), truncated-HKSJ, and fixed-effects methods, with a Bayesian sensitivity analysis. A pre-specified subgroup contrasted septic shock with sepsis without shock. This review was registered in PROSPERO (CRD420261334243). **Results:** Eight RCTs (*n* = 3707) provided the data; all administered 20% albumin. Overall, albumin was not robustly associated with reduced mortality (RR 0.94; I^2^ = 0%), significant only under the pre-registered HKSJ correction (0.91–0.98) but null under the standard, truncated-HKSJ, and fixed-effects models (0.87–1.03, *p* = 0.17), identifying this as a low-heterogeneity artifact. In septic shock (k = 6), the effect was larger and consistent (RR 0.90, 0.82–0.99, *p* = 0.03), with no benefit in sepsis without shock (RR 1.10; interaction *p* = 0.08). The certainty was low (GRADE). **Conclusions:** In adults with sepsis, hyperoncotic albumin was not associated with a statistically significant reduction in all-cause mortality, and the certainty of evidence was low (GRADE). This is consistent with the 2026 Surviving Sepsis Campaign guidelines, which permit case-by-case use of albumin in this setting but do not recommend its routine use. Adequately powered randomized trials are required.

## 1. Introduction

Sepsis is defined as life-threatening organ dysfunction caused by a dysregulated host response to infection and is among the foremost causes of death in critically ill patients worldwide [1,2]. Global epidemiological data from the 2017 Global Burden of Disease Study show an estimated 48.9 million incident cases and 11.0 million sepsis-related deaths, corresponding to approximately 19.7% of all global mortality [3,4]. In recognition of this burden, the World Health Organization declared sepsis a global health priority in 2017 [5]. Despite decades of progress in intensive care, sepsis-related case fatality remains > 20%, and no pharmacological intervention has demonstrated a robust and consistent survival benefit across the full spectrum of disease severity [3,6].

Human albumin, the most abundant plasma protein, has long been a candidate for resuscitation and supplementation in sepsis, justified by both oncotic and pleiotropic non-oncotic properties [7]. In addition to maintaining colloid osmotic pressure and expanding the intravascular compartment, albumin stabilizes the endothelial glycocalyx, attenuates capillary leakage, exerts antioxidant activity through reactive free thiol groups, modulates the transport of inflammatory mediators, and buffers nitric oxide bioavailability [8,9]. These mechanisms provide a biological rationale for the preferential use of hyperoncotic solutions at concentrations of 20% or 25% in hemodynamic instability, endothelial injury, and profound capillary leak that characterize septic shock [10]. Hypoalbuminemia, a near-universal feature of critical illness, is independently associated with increased mortality and organ dysfunction, providing further pathophysiological support for albumin-supplementation strategies [11].

Current international guidelines reflect this biological plausibility but have moved against albumin over successive editions while continuing to qualify their recommendations in a way that signals the limited certainty of the available evidence. The Surviving Sepsis Campaign reversed its position between editions: the 2021 guidelines issued a weak, conditional recommendation for albumin in patients who receive substantial volumes of crystalloids [6], whereas the 2026 edition suggests crystalloids alone over crystalloids with supplemental albumin (conditional recommendation, moderate certainty of evidence) while explicitly permitting supplemental albumin on a case-by-case basis in patients who have already received large crystalloid volumes and in patients with cirrhosis and sepsis [12]. Concordantly, the European Society of Intensive Care Medicine (ESICM) resuscitation-fluid guidelines conditionally recommend crystalloids rather than albumin in critically ill patients and in sepsis, specifically, endorsing albumin only in cirrhosis [13]. The International Collaboration for Transfusion Medicine Guidelines similarly found few evidence-based indications for routine albumin use in critical care, with conditional recommendations restricted to cirrhosis-related complications [14]. A recent synthesis of expert opinions confirmed the predominance of crystalloids as first-line therapy, with albumin occupying a supplementary role in selected patients [15]. This convergence on conditional recommendations, all of which retain explicit exceptions, underscores that mortality—the endpoint that governs recommendation strength—remains the critical unresolved question [16].

Several randomized controlled trials (RCTs) have examined the use of hyperoncotic albumin in sepsis. The largest study, the Albumin Italian Outcome Sepsis (ALBIOS) (*n* = 1818), found no significant reduction in 28- or 90-day mortality with 20% albumin versus crystalloids, although a post hoc subgroup analysis suggested a survival benefit restricted to patients with septic shock (relative risk [RR] 0.87, 95% CI 0.77–0.99), with no benefit in sepsis without shock (RR 1.13, 95% CI 0.92–1.39; test for interaction *p* = 0.03) [17]. The recently published Albumin Replacement Therapy in Septic Shock (ARISS) trial (*n* = 440), designed for septic shock, found no significant reduction in 90-day mortality (43.3 vs. 45.9%; RR 0.94, 95% CI 0.76–1.17; *p* = 0.71) but was terminated early for slow recruitment and was substantially underpowered relative to its planned enrolment of 1662 patients, with additional dilution from a 48.6% rate of albumin administration in the control arm [18].

Prior systematic reviews have attempted to synthesize this evidence, including those by Bannard-Smith et al. [19] and Bai et al. [20]. Both share methodological limitations relevant to the present study. Their searches predated the 2025–2026 trials, and neither restricted the comparators exclusively to crystalloids. Bannard-Smith et al. included a trial in which hydroxyethyl starch served as the primary comparator [21], a synthetic colloid contraindicated in sepsis based on increased mortality and acute kidney injury in large trials [22] and, ultimately, suspended from the market in 2022 [23]. A concurrent dual frequentist–Bayesian meta-analysis restricted to septic shock by Mendes et al. [24] reported a 10% relative risk reduction (RR 0.90, 95% CI 0.83–0.99), pooling both hyperoncotic and iso-oncotic albumin and relying substantially on the extracted subgroup strata. These related reviews did not isolate the question addressed here: none restricted the intervention to hyperoncotic (≥20%) albumin and the comparator exclusively to crystalloids. The present review adds this specific contrast to the existing evidence.

Therefore, the present systematic review and meta-analysis was undertaken to provide an up-to-date synthesis of the mortality evidence through March 2026, incorporating all eligible RCTs that compared hyperoncotic albumin (≥20%) exclusively against crystalloids in adults with sepsis or septic shock. We placed particular emphasis on the transparent reporting of every statistical consideration, including the sensitivity of the pooled estimate to the variance estimation method, and on a pre-specified evaluation of whether any benefit is concentrated in septic shock, the population for which current guidelines conditionally favor crystalloids alone while still allowing for albumin on a case-by-case basis and for which the mortality evidence, therefore, remains decision relevant.

## 2. Materials and Methods

### 2.1. Protocol and Registration

This systematic review and meta-analysis was conducted and reported in accordance with the Preferred Reporting Items for Systematic Reviews and Meta-Analyses (PRISMA) 2020 statement [25] (completed checklist provided in Supplementary File S4) and, where feasible, the Cochrane Handbook for Systematic Reviews of Interventions [26]. Screening, data extraction, and risk-of-bias assessment were performed by a single reviewer with independent verification by a second reviewer rather than by full independent duplication; these steps deviate from the Cochrane Handbook recommendations. The protocol was prospectively registered with PROSPERO (CRD420261334243).

### 2.2. Eligibility Criteria

Eligibility was defined using the PICOS framework (Participants, Intervention, Comparator, Outcomes, Study design). Participants were adults (≥18 years) with sepsis or septic shock according to any consensus definition in use at the time of the trial, including criteria predating Sepsis-3 (2016) [1], to avoid excluding trials of established merit based on a retrospectively applied definitional revision. No restrictions were placed on the setting, region, or period.

The eligible intervention was intravenous hyperoncotic albumin at a concentration ≥ 20%, irrespective of dose, frequency, or duration. The eligible comparator was intravenous crystalloids only (isotonic saline, balanced crystalloids including Ringer’s lactate, Ringer’s acetate, Plasmalyte, or a combination thereof). Trials in which the control arm received any colloid, including iso-oncotic (4–5%) albumin, hydroxyethyl starch, gelatin, or dextran, as the primary comparator were excluded because such comparisons do not reflect standard resuscitation practice and preclude the interpretation of effects attributable to hyperoncotic albumin specifically.

Eligible outcomes required the reporting of all-cause mortality at any time point. The primary outcome was all-cause mortality at the longest available follow-up period reported in each trial. The pre-specified secondary outcomes were all-cause mortality at 90 days, 28 days, and intensive care unit (ICU) or hospital discharge. Eligible designs were parallel-group RCTs; quasi-randomized, cluster-randomized, crossover, and observational studies were excluded. No restrictions were applied to sample size, language, or blinding. Both peer-reviewed publications and congress abstracts were eligible, provided that sufficient methodological details were available for risk-of-bias assessment; non-peer-reviewed preprints were excluded.

### 2.3. Information Sources and Search Strategy

The search for studies published before 21 March 2024, was adopted from Bai et al. [20], who searched MEDLINE (via PubMed), EMBASE, the Cochrane Central Register of Controlled Trials (CENTRAL), ClinicalTrials.gov, and the WHO International Clinical Trials Registry Platform. An independent update search was conducted on 10 March 2026, covering 21 March 2024 to 10 March 2026, in MEDLINE (via PubMed), EMBASE, and Cochrane CENTRAL; the search strings are provided in Supplementary File S1, which also documents that the update search reproduced the databases and search logic of Bai et al. [20] for the interval from 21 March 2024, ensuring seamless continuity with the adopted baseline search. No language restrictions were imposed. Reference lists were not hand-searched. ClinicalTrials.gov was consulted to ascertain the status and availability of the results of the ALBIOSS-BALANCED trial (NCT03654001) [27].

### 2.4. Study Selection

Titles and abstracts from the updated search were screened by one reviewer (C.J.W.), with a 20% random sample independently co-screened by a second reviewer to confirm reliability (G.T.); all potentially eligible records were retrieved in full text. Full-text screening against the eligibility criteria was performed by one reviewer (C.J.W.) and verified by a second reviewer (M.J.). Disagreements were resolved through discussion. The process is illustrated in a PRISMA 2020 flow diagram.

### 2.5. Data Extraction

Data were extracted using a piloted form by one reviewer (C.J.W.) and independently verified by a second reviewer (A.Z.); disagreements were resolved by consensus. If a required characteristic was not reported, it was recorded as such. Dong et al. specified a dose of 20 g of human albumin but did not state the concentration. An inquiry sent to the corresponding author (March 2026) received no response, and a 20% solution was assumed based on the standard hyperoncotic formulation available in the study setting. Accordingly, this trial was subjected to a pre-registered sensitivity analysis. For ALBIOS, the septic-shock stratum used in the subgroup analysis (1121 patients with septic shock versus 660 without; 90-day mortality relative risk 0.87, 95% CI 0.77–0.99) was taken from the post hoc subgroup analysis reported in the Results section and Supplementary Figure S3 of the original publication of Caironi et al. [17]. For ICARUS-ED [28], the refractory-hypotension stratum (*n* = 386; 30-day mortality of 35/197 with albumin versus 30/189 with crystalloids) was extracted from Supplementary Table S6 of the online supplement. If a trial enrolled a mixed sepsis population and reported a separately extractable septic-shock stratum, that stratum was used for the septic-shock subgroup analysis.

### 2.6. Risk of Bias Assessment

The risk of bias for the primary outcome was assessed using the Cochrane Risk of Bias tool version 2 (RoB 2) [29] across the five standard domains by one reviewer (C.J.W.) and verified by a second reviewer (G.T.). The results are presented as a traffic-light plot and a domain-level summary table.

### 2.7. Statistical Analysis

Analyses were performed using R (version 4.5.1) with the metafor package (version 4.8.0) [30]. The effect measure for all mortality outcomes was the risk ratio (RR) with a 95% confidence interval (CI). A random-effects inverse-variance model was applied throughout, with the between-study variance (τ^2^) estimated using the restricted maximum likelihood (REML) [31]. Heterogeneity was quantified using the Q statistic, I^2^, τ^2^, and 95% prediction intervals [32].

The primary analysis applied the Hartung–Knapp–Sidik–Jonkman (HKSJ) correction to the confidence intervals, as pre-registered [33]. Because the observed between-study heterogeneity proved negligible (I^2^ = 0%), a condition under which the HKSJ correction can generate confidence intervals narrower than the uncorrected model and thereby overstate precision, three further variance specifications were added post hoc to make the inference fully transparent: the standard z-based random-effects interval; a truncated HKSJ interval in which the ad-hoc variance factor is constrained to be ≥1 [34]; and a fixed-effects interval. As an additional post hoc sensitivity analysis, a Bayesian random-effects meta-analysis was performed with a weakly informative prior on the pooled log risk ratio (normal, mean of 0, and standard deviation of 0.5); the posterior median RR, 95% credible interval, and posterior probabilities of any benefit (RR < 1.0) and of a ≥5% relative reduction (RR < 0.95) are reported. These post hoc additions are described in Section 2.9.

#### 2.7.1. Subgroup Analyses

Two subgroup analyses were pre-specified for the primary outcomes. First, disease severity at enrolment was examined by contrasting septic shock with sepsis without shock. Trials enrolling exclusively patients with septic shock, together with the separately extractable septic-shock stratum of ALBIOS, formed the septic-shock subgroup, while the complementary non-shock strata of ALBIOS and trials of severe sepsis without shock formed the comparator subgroup. Subgroup differences were tested using a mixed-effects (moderator) model. Where a trial enrolled a mixed population, the separately extractable septic-shock stratum was used for the septic-shock subgroup (the septic-shock stratum of ALBIOS [17] and the refractory-hypotension stratum of ICARUS-ED [28]). Two gradations were examined: restriction to trials with a whole-trial or pre-specified septic shock population (excluding the post hoc ICARUS-ED refractory-hypotension stratum) and restriction to non-cirrhotic septic shock. Second, the presence of cirrhosis was examined by comparing trials enrolling patients with cirrhosis or acute-on-chronic liver failure with those that did not.

#### 2.7.2. Sensitivity Analyses

The following five sensitivity analyses were pre-specified for the primary outcome: restriction to trials with ≥50 participants per arm; restriction to large RCTs (≥400 participants in total); exclusion of Dong et al. [35] on account of their combination-intervention design (20% albumin added to crystalloids); exclusion of ALPS [36] (cirrhosis-specific population); and a leave-one-out analysis.

#### 2.7.3. Publication Bias

Publication bias was assessed visually using a funnel plot and formally using Egger’s regression test [37]. Because fewer than ten trials contributed to the primary analysis, Egger’s test is reported with the explicit caveat that its statistical power is limited to this number of studies.

### 2.8. Certainty of Evidence

The certainty of evidence for the primary and subgroup outcomes was rated using the GRADE framework [16] across risk of bias, inconsistency, indirectness, imprecision, and publication bias, and summarized in a Summary of Findings table.

### 2.9. Deviations from the Registered Protocol

The following material amendments to the registered protocol (PROSPERO CRD420261334243) were declared: (1) The pre-registered primary analysis specified only the HKSJ correction; because heterogeneity proved negligible and the HKSJ interval was consequently anticonservative, three further variance specifications (standard z-based, truncated HKSJ, and fixed effects) and a Bayesian sensitivity analysis were added post hoc to characterize the robustness of the inference. (2) The severity subgroup was pre-registered at the study level (septic shock versus general sepsis); it was implemented at the stratum level, using the separately extractable septic-shock and non-shock strata of ALBIOS and ICARUS-ED, with a restriction to non-cirrhotic septic shock added as gradation. For ICARUS-ED, the refractory-hypotension stratum (systolic blood pressure < 90 mmHg after a fluid challenge of ≥1000 mL) served as the septic-shock stratum, and its mortality counts were verified against the trial’s published online supplement (Supplementary Table S6 of Ref. [28]). (3) The cirrhosis subgroup was pre-registered as ALPS versus all other trials; Goyal & Agarwal was additionally assigned to the cirrhosis stratum, as that trial also enrolled exclusively cirrhotic patients. (4) Contrary to the registered statement that authors would not be contacted, an inquiry was sent to the corresponding author of Dong et al. [35] regarding the albumin concentration, which was not stated in the publication; no response was received, so the assumed 20% concentration remains unconfirmed, a limitation addressed by the pre-registered sensitivity analysis excluding this trial. (5) The pre-registered synthesis specified a Mantel–Haenszel random-effects model implemented in the R meta package; the analysis, instead, used an inverse-variance random-effects model with restricted maximum likelihood estimation of τ^2^ in the metafor package, the specification that accommodates the truncated-HKSJ and additional variance methods reported here. Given the negligible heterogeneity (I^2^ = 0%), the two models yield effectively identical study weights, and the pooled estimate is unchanged. Two minor points are also noted: mortality was extracted from the analyzed populations, which for ALBIOS [17] and ARISS [18] were marginally smaller than the randomized populations owing to missing follow-up; and Egger’s test, pre-registered for k ≥ 10 [37], is reported as a supportive analysis at k = 8 with explicit acknowledgement of limited power. The eligibility criteria, primary and secondary outcomes, and five pre-registered sensitivity analyses were applied as registered.

## 3. Results

### 3.1. Study Selection

**Figure 1:**
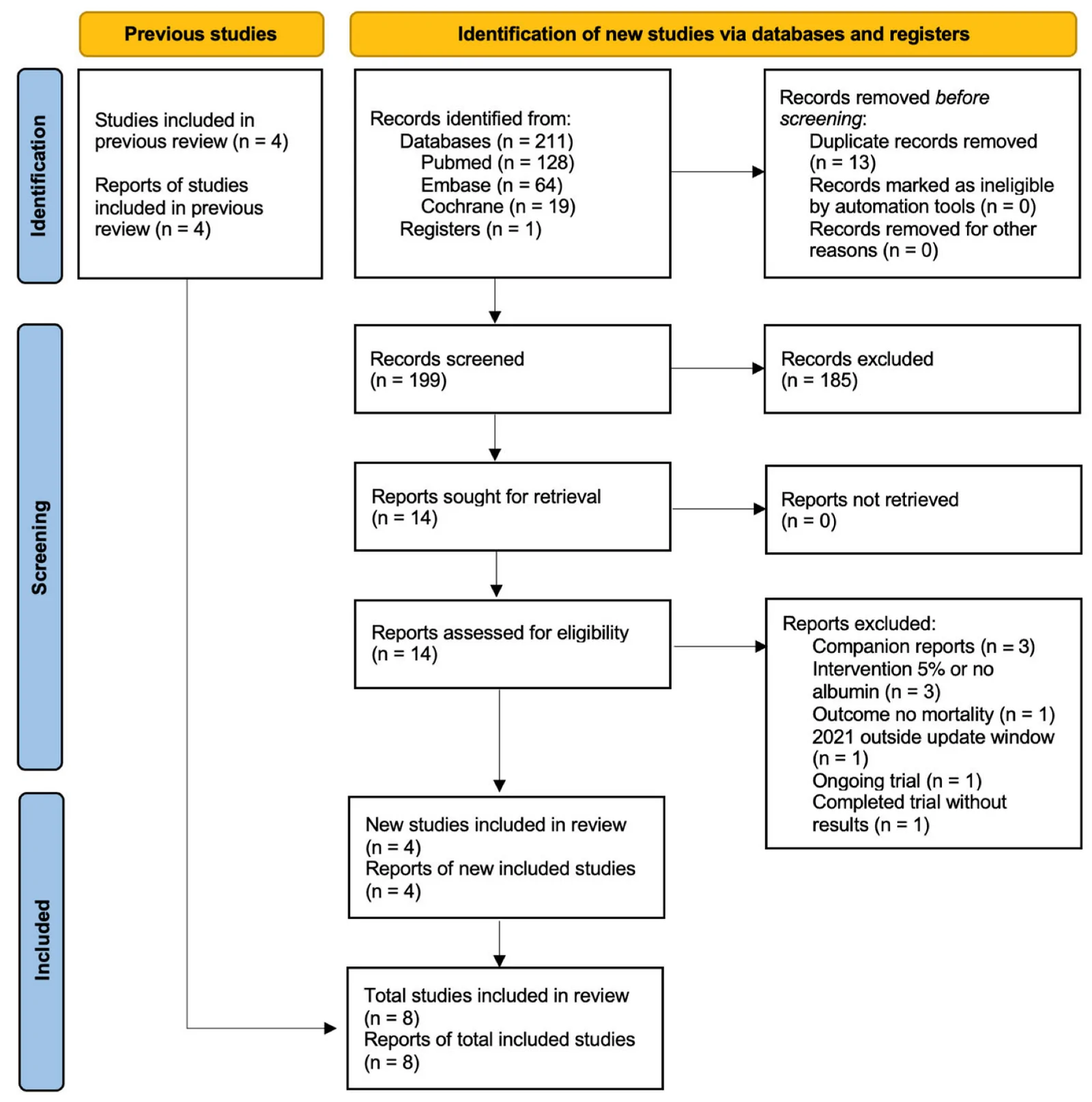
PRISMA 2020 flow diagram of the updated systematic review. The updated search covered 21 March 2024 to 10 March 2026 and was combined with the eligible studies identified by Bai et al. [20].

The selection process is illustrated in Figure 1. The updated search (21 March 2024 to 10 March 2026) retrieved 211 records from the databases (128 MEDLINE, 64 EMBASE, and 19 CENTRAL), and one additional trial was identified through the ClinicalTrials.gov register (ALBIOSS-BALANCED, NCT03654001) [27], yielding a total of 212 records. After the removal of 13 duplicates, 199 records were screened based on their titles and abstracts, of which 185 were excluded and 14 reports were sought for retrieval. After consolidating three companion reports of the same study, these corresponded to ten unique database studies and a single registry report. Four newly published trials met all eligibility criteria—ICARUS-ED (Intervention with Concentrated Albumin for Undifferentiated Sepsis in the Emergency Department) [28], Goyal & Agarwal [38], Dong et al. [35] and ARISS [18]—and were combined with the four eligible trials identified by Bai et al. [20] (ALBIOS [17], EARSS (Early Albumin Resuscitation during Septic Shock) [39], Kakaei et al. [40] and ALPS (Albumin versus Crystalloid in Cirrhotics with Septic Shock) [36]). Ten reports were excluded at the full-text stage, as follows: three companion reports of studies already listed; ABC (Albumin versus Balanced Crystalloid)-Sepsis (Gray et al. [41]), which administered iso-oncotic 5% albumin rather than a hyperoncotic (≥20%) solution; Gupta et al. [42], a comparison of vasopressors (terlipressin versus noradrenaline) rather than albumin; Maiwall et al. [43] (TOP-204), whose comparator combined 5% and 20% albumin rather than crystalloids alone; Verma et al. [44], a 2021 conference abstract falling outside the update-search window; the microcirculation trial of Cusack et al. [45], which reported only a microcirculatory surrogate with no time-defined mortality endpoint; the ALBUS (Albumin Use in Surgical Septic Shock) trial (NCT07212582) [46], an ongoing study with no outcome data; and the completed but unpublished ALBIOSS-BALANCED trial (NCT03654001) [27], whose results had not been presented or published at the time of analysis (author communication, P. Caironi, July 2026). Eight RCTs contributed extractable all-cause mortality data to the quantitative synthesis [17,18,28,35,36,38,39,40]; the full-text inclusion and exclusion decisions with reasons are detailed in Supplementary Table S3.

### 3.2. Characteristics of Included Studies

The eight included RCTs were published between 2011 and 2026 and enrolled 3707 patients who contributed to the primary analysis (1863 in the albumin arm and 1844 in the crystalloid arm). The analyzed sample sizes per arm ranged from 10 [40] to 888 (ALBIOS, at the 90-day time point [17]). Trials have been conducted across Europe [17,18,39], the Middle East [40], Asia [36,37,38], and Oceania [28]. All patients received 20% albumin; none received 25% albumin exclusively. The crystalloid comparator comprised isotonic saline, balanced crystalloids, or Plasmalyte. Sepsis was defined by the pre-Sepsis-3 criteria in earlier trials [17,39,40] and by Sepsis-3 thereafter. Four trials exclusively enrolled patients with septic shock (EARSS [39], ALPS [36], Goyal & Agarwal [38], and ARISS [18]); three enrolled a mixed population of sepsis and septic shock (ALBIOS [17], Dong et al. [35], and ICARUS-ED [28]); and one enrolled patients with severe sepsis without a shock requirement (Kakaei et al. [40]). Two trials were conducted specifically in patients with cirrhosis or acute-on-chronic liver failure (ALPS [36] and Goyal & Agarwal [38]), while Dong et al. [35] enrolled an unselected sepsis/septic-shock ICU population and did not require cirrhosis. The full characteristics are summarized in Table 1 (design and population) and Table 2 (interventions, comparators, and mortality outcomes).

### 3.3. Risk of Bias

**Figure 2:**
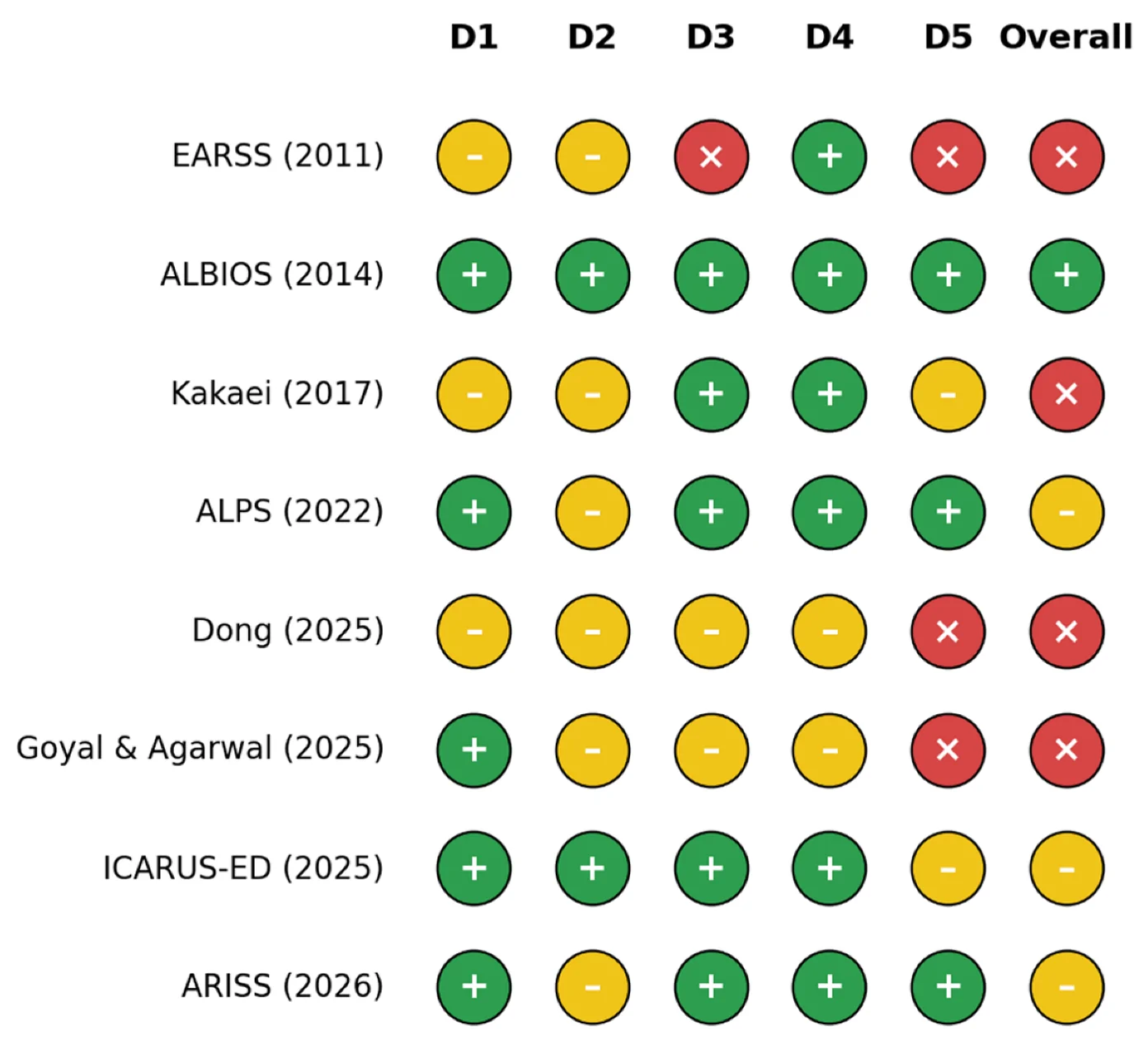
Risk-of-bias assessment (Cochrane RoB 2) for the primary outcome (all-cause mortality at the longest available follow-up) across the eight included trials. Domains: D1, bias arising from the randomization process; D2, bias due to deviations from intended interventions; D3, bias due to missing outcome data; D4, bias in measurement of the outcome; and D5, bias in selection of the reported result. The judgement for each domain is shown by both a symbol and a colour: a green plus sign (+) denotes low risk; an amber minus sign (−), some concerns; and a red cross (×), high risk. The “Overall” column gives the trial-level risk-of-bias judgement across all five domains. References: EARSS, 2011 [39]; ALBIOS, 2014 [17]; Kakaei et al., 2017 [40]; ALPS, 2022 [36]; Dong et al., 2025 [35]; Goyal & Agarwal, 2025 [38]; ICARUS-ED, 2025 [28]; ARISS, 2026 [18].

The largest trials, ALBIOS [17] and ARISS [18], were rated as low or low-to-moderate risk across most domains, reflecting centralized randomization, prospective registration, and pre-specified analysis plans. None of the included trials blinded the participants or clinicians, given the impracticability of blinding fluid interventions; outcome assessment for all-cause mortality is objective and was rated as low risk. Kakaei et al. [40] and Goyal & Agarwal [38] were rated as having some concerns owing to small size and limited reporting; and ARISS [18] carried a domain concern from the 48.6% rate of albumin use in the control arm. Domain-level ratings are provided in Supplementary Table S2 and Figure 2.

### 3.4. Primary Analysis: All-Cause Mortality at Longest Available Follow-Up

Across the eight trials (*n* = 3707), hyperoncotic albumin was associated with a pooled RR of 0.94 for all-cause mortality, with negligible heterogeneity (I^2^ = 0%; τ^2^ = 0). Because heterogeneity was absent, the inference depended materially on the variance estimation method, and we report the pre-registered HKSJ analysis together with three post hoc variance specifications (Table 3). Under the pre-registered HKSJ correction, the 95% CI excluded the null (0.91–0.98, *p* = 0.01). However, because the ad hoc HKSJ variance factor fell well below unity (H = 0.16) under the observed homogeneity, this interval reflects a recognized artifact of the HKSJ estimator at low heterogeneity rather than genuine evidential strength. Because the observed heterogeneity was essentially zero, the HKSJ correction paradoxically produced a narrower confidence interval than the standard model, a known limitation of this method at very low heterogeneity, and this does not represent a genuine increase in statistical power. The post hoc variance specifications all returned the interval across the null: the standard z-based and fixed-effects models were identical (0.87–1.03, *p* = 0.17), and the truncated HKSJ resulted in 0.85–1.04 (*p* = 0.21). Therefore, we interpret the overall effect as directionally favorable but not robustly significant, and we do not regard the pre-registered HKSJ result as establishing a mortality benefit in the overall sepsis population.

**Figure 3:**
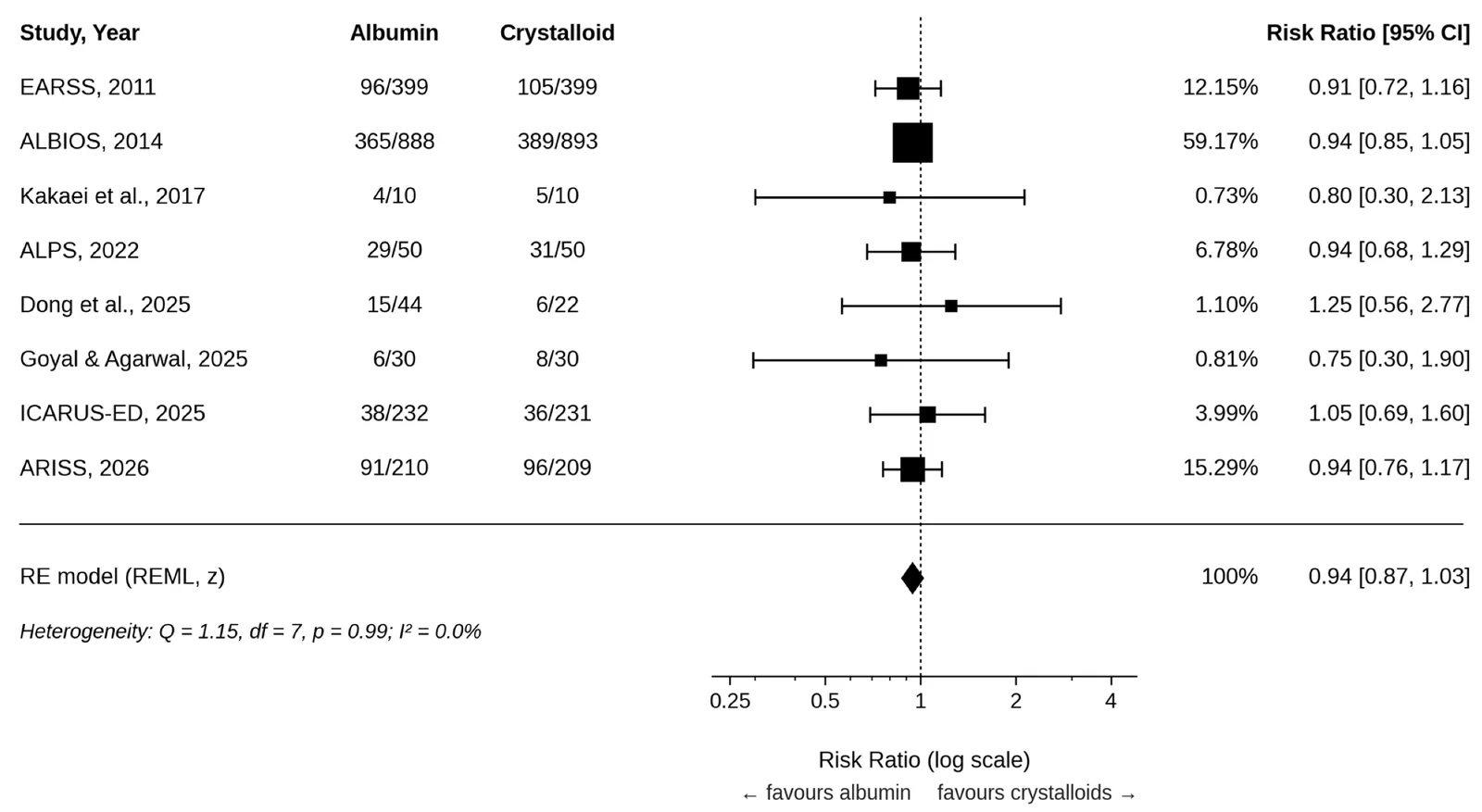
Forest plot of all-cause mortality at the longest available follow-up for hyperoncotic albumin (≥20%) versus crystalloids across the eight included trials. Study-level risk ratios (RRs) with 95% confidence intervals are shown with the random-effects pooled estimate (RR 0.94; I^2^ = 0%). The pooled estimate is from the standard random-effects (z-based) model; see Table 3 for results under the alternative variance-estimation methods. A risk ratio below 1 favors albumin. References: EARSS, 2011 [39]; ALBIOS, 2014 [17]; Kakaei et al., 2017 [40]; ALPS, 2022 [36]; Dong et al., 2025 [35]; Goyal & Agarwal, 2025 [38]; ICARUS-ED, 2025 [28]; and ARISS, 2026 [18]. CI, confidence interval; RE, random effects; REML, restricted maximum likelihood.

The Bayesian sensitivity analysis (weakly informative prior) yielded a posterior median RR of 0.94 (95% credible interval 0.87–1.03), with a posterior probability of any mortality benefit (RR < 1.0) of 91.3% and a ≥5% relative reduction (RR < 0.95) of 56.1%. The forest plot is shown in Figure 3.

Because heterogeneity was absent, the pooled interval was method-dependent and is reported under four variance estimation specifications in Table 3.

### 3.5. Secondary Analyses

At 90 days (k = 4; ALBIOS [17], Kakaei [40], Dong [35], and ARISS [18]), the pooled RR was 0.95 (95% CI 0.86–1.04; *p* = 0.25; I^2^ = 0%). At 28 days (k = 6), the pooled RR was 0.95 (95% CI 0.85–1.05; *p* = 0.28; I^2^ = 0%). Both secondary estimates were directionally concordant with the primary analysis and individually non-significant, consistent with the primary trial results of ALBIOS [17] and ARISS [18].

### 3.6. Subgroup Analyses

#### 3.6.1. Septic Shock vs. Sepsis Without Shock

**Figure 4:**
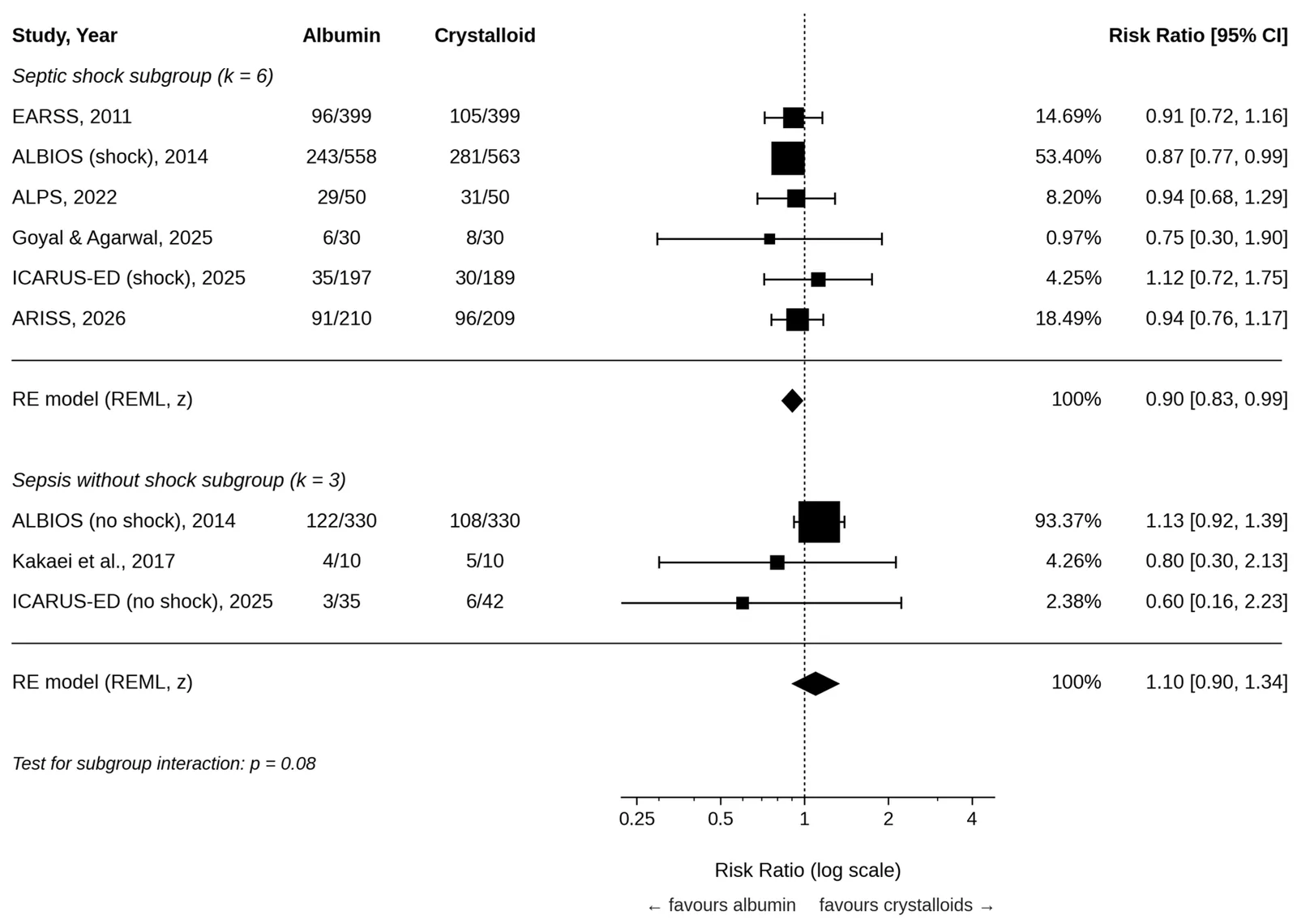
Forest plot of all-cause mortality by disease severity: septic shock versus sepsis without shock. The septic-shock subgroup (k = 6) comprises the four exclusively septic-shock trials (EARSS [39], ALPS [36], Goyal & Agarwal [38], ARISS [18]) and the separately extractable septic-shock strata of ALBIOS [17] and ICARUS-ED [28]; the sepsis-without-shock subgroup comprises the non-shock strata of ALBIOS [17] and ICARUS-ED [28] and the severe-sepsis trial of Kakaei et al. [40]. Study-level risk ratios (RR) with 95% confidence intervals and the subgroup random-effects pooled estimates (septic shock: RR 0.90, 95% CI 0.82–0.99; sepsis without shock: RR 1.10, 95% CI 0.90–1.34) are shown, together with the test for subgroup interaction (*p* = 0.08); a risk ratio below 1 favors albumin. The ALBIOS and non-shock strata were incorporated as detailed in Supplementary File S5. CI, confidence interval; RE, random effects; REML, restricted maximum likelihood.

In the pre-specified severity subgroup analysis, hyperoncotic albumin showed a larger and directionally consistent effect in patients with septic shock. Pooling the four exclusively septic-shock trials with the extractable septic-shock strata of ALBIOS [17] and ICARUS-ED [28] (k = 6; *n* = 2884), the RR was 0.90 (standard 95% CI 0.82–0.99; *p* = 0.03; raw HKSJ 0.84–0.97, *p* = 0.01; truncated HKSJ 0.80–1.02, *p* = 0.09; I^2^ = 0%) (Figure 4). In contrast, sepsis without shock (the non-shock strata of ALBIOS [17] (RR 1.13) and ICARUS-ED [28], together with the severe sepsis trial of Kakaei et al. [40]) showed no benefit (pooled RR 1.10, 95% CI 0.90–1.34; *p* = 0.36). The test for subgroup differences approached significance (*p* = 0.08). The within-trial gradient in ALBIOS [17] is illustrative: the full trial produced an RR of 0.94, the septic-shock stratum an RR of 0.87, and the non-shock stratum an RR of 1.13.

Two gradations confirmed the robustness of the septic-shock signal. The ICARUS-ED [28] contribution was its refractory-hypotension stratum (systolic blood pressure < 90 mmHg after a ≥1000 mL fluid challenge; 30-day mortality 35/197 with albumin versus 30/189 with crystalloids, RR 1.12), the only septic-shock estimate to favor the comparator; these counts were verified against the trial’s published online supplement (Supplementary Table S6 of Ref. [28]). Restriction to the five trials with a whole-trial or pre-specified septic-shock population (i.e., excluding this single post hoc emergency-department stratum) resulted in a marginally stronger estimate (k = 5; RR 0.90, standard 95% CI 0.82–0.98; *p* = 0.02). Restriction to non-cirrhotic septic shock (EARSS [39], ALBIOS-shock [17], and ARISS [18]; k = 3) produced an RR of 0.89 (standard 95% CI 0.81–0.99; *p* = 0.03), indicating that the signal is not driven by cirrhosis-specific trials. Across all definitions, the septic-shock point estimate remained between 0.89 and 0.91 and reached significance under the standard random-effects model, while remaining borderline under the most conservative small-sample correction.

#### 3.6.2. Cirrhosis vs. Non-Cirrhosis

Trials enrolling patients with cirrhosis or acute-on-chronic liver failure (ALPS [36] and Goyal & Agarwal [38]; k = 2) yielded a pooled RR of 0.91 (95% CI 0.39–2.17), while the non-cirrhosis trials (k = 6) yielded an RR of 0.95 (95% CI 0.90–0.99). Given the small number of cirrhosis trials and the distinct pathophysiology of albumin deficiency in cirrhosis, this comparison was exploratory.

### 3.7. Sensitivity Analyses

The primary point estimate was stable across all five pre-registered sensitivity analyses (RR 0.94–0.95) (Table 4). Restriction to trials with ≥50 participants per arm (k = 5) resulted in an RR of 0.94 (0.91–0.98); restriction to large RCTs with ≥400 participants in total (k = 4) produced 0.94 (0.90–0.99); exclusion of Dong et al. [35] for its combination-intervention design (k = 7) resulted in 0.94 (0.91–0.97); exclusion of ALPS [36] (k = 7) resulted in 0.94 (0.90–0.99); and leave-one-out estimates ranged from 0.94 to 0.95 with I^2^ = 0% throughout and omission of ALBIOS [17] producing the widest interval (0.88–1.01). As for the primary analysis, the confidence intervals in these HKSJ-based sensitivity analyses are subject to the low-heterogeneity caveat; the invariant point estimate, rather than the interval width, is a robust finding.

### 3.8. Publication Bias

**Figure 5:**
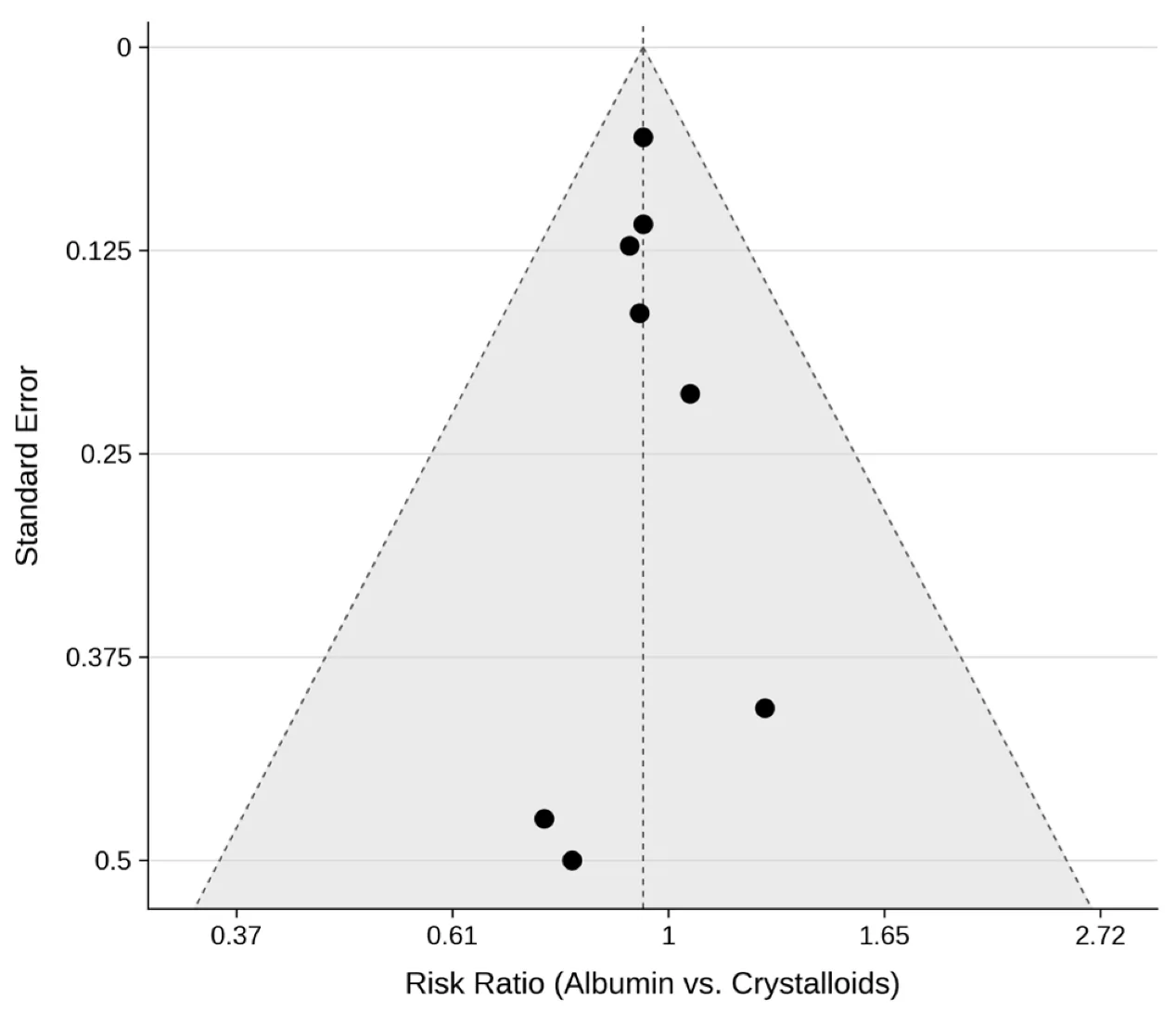
Funnel plot of the eight trials contributing to the primary outcome, plotting study-level risk ratios (log scale) against their standard errors. Each dot represents one included trial. The vertical dashed line indicates the pooled random-effects estimate (RR 0.94), and the shaded grey region denotes the 95% pseudo-confidence region expected in the absence of publication bias and between-study heterogeneity (pooled estimate ± 1.96 × standard error); the diagonal dashed lines mark its boarders. Symmetry of the trials about the pooled estimate argues against small-study effects.

Visual inspection of the funnel plot (Figure 5) revealed no significant asymmetry. Egger’s regression test was non-significant (intercept 0.03; t = 0.10; *p* = 0.92). This result should be interpreted with caution, as the power of Egger’s test was limited to eight trials.

### 3.9. Certainty of Evidence and Protocol Deviations

The certainty of evidence for the primary outcome was rated low under GRADE, downgraded for imprecision (the 95% CI is compatible with a clinically relevant benefit, no effect, and potential harm), and for indirectness (contribution of extracted strata and mixed-severity populations). The septic-shock subgroup was also rated low. The summary of findings is presented in Table 5.

## 4. Discussion

This updated meta-analysis, restricted to hyperoncotic albumin (≥20%) against a strictly crystalloid comparator, yielded two findings that are complementary rather than contradictory. In the overall sepsis population, hyperoncotic albumin did not robustly reduce all-cause mortality; the point estimate was consistently favorable (RR 0.94), but its confidence interval crossed the null under every variance method, except the uncorrected HKSJ estimator, whose apparent significance is an artifact of near-zero heterogeneity rather than a genuine strengthening of the evidence. In the pre-specified septic-shock subgroup, by contrast, the effect was larger (RR 0.90), internally consistent across trials and gradations, and statistically significant under the standard random-effects model, while no benefit was observed in sepsis without shock (RR 1.10, non-significant).

In our view, transparent reporting of the variance-method dependence is a methodological obligation rather than a technical aside. The Hartung–Knapp–Sidik–Jonkman correction is widely and appropriately recommended to guard against overly narrow intervals when few trials are pooled [33]; however, when the observed heterogeneity is below chance expectation, its ad hoc variance factor falls below unity, and the correction paradoxically narrows the interval, generating spurious precision. Constraining the factor to at least unity removes this artifact. Reporting the pooled estimate under the standard, raw-HKSJ, truncated-HKSJ, and fixed-effects models, alongside a Bayesian posterior, allows the reader to see that the point estimate is entirely stable and that only the interval—and, therefore, the binary verdict of significance—moves with the method. We regard the truncated HKSJ and standard estimates as the honest primary inference for the overall population, and we do not claim overall significance.

The concentration of the effect in septic shock is biologically coherent. Endothelial injury, glycocalyx degradation, vasoplegia, and capillary leak, which albumin is proposed to counter, are most pronounced in shock, where the intravascular persistence and endothelial-stabilizing properties of a hyperoncotic solution would be expected to matter most [8,9,10]. The within-trial gradient in ALBIOS [17]—no benefit overall, a significant benefit in the septic-shock stratum, and no benefit in the non-shock stratum (RR 1.13)—recapitulates this severity dependence in a single dataset, and the pooled subgroup analysis extends it across trials. This pattern also clarifies why the overall estimate is attenuated: pooling non-shock patients, in whom albumin confers no benefit, dilutes the real effect that is present in shock.

These results meta-analytically corroborate, rather than overturn, earlier pro-albumin syntheses. Bai et al. [20] reported a septic shock RR of 0.91 (0.84–0.99), and the concurrent septic-shock meta-analysis of Mendes et al. [24] reported an RR of 0.90 (0.83–0.99); our septic-shock estimate of 0.90 is essentially identical, despite differing inclusion criteria, comparator restriction, and use of full-trial rather than subgroup data. The convergence of three independently conducted analyses on a ~10% relative risk reduction in septic shock is more persuasive than any single estimate. Notably, our analysis differs from that of Mendes et al. [24] in two respects that strengthen the interpretation: we restricted the comparator to crystalloids and the concentration to hyperoncotic solutions, isolating the effect of 20% albumin against standard resuscitation, and we relied on full-trial data supplemented by extractable septic-shock strata rather than predominantly on subgroups. The concordance of the estimates across these methodological differences indicates that the septic-shock signal is not an artifact of any analytical choice.

Our findings also place the individually inconclusive ARISS [18] trial in this context. ARISS was neutral and formally underpowered, terminated at 440 of a planned 1662 patients, and further diluted by a 48.6% rate of albumin administration in its control arm. Interpreted in isolation, these results indicate a negative trial. Interpreted within the pooled septic shock evidence, its point estimates (RRs of 0.94 at 90 days and 0.81 at 28 days) are fully consistent with the pooled RR of 0.90 and contribute to, rather than contradict, the aggregate signal. A single underpowered trial with substantial crossover is expected to yield a non-significant estimate even when a true effect of this magnitude exists; meta-analysis is precisely the instrument that recovers such an effect.

Taken together, these observations bear directly on the position adopted by recent guidelines. The 2026 Surviving Sepsis Campaign suggests crystalloids alone over crystalloids with supplemental albumin but qualifies this conditional recommendation by permitting albumin on a case-by-case basis in patients who have already received large crystalloid volumes and in patients with cirrhosis and sepsis [12], reversing the weak recommendation in favor of albumin made in 2021 [6]. The 2026 Surviving Sepsis Campaign guidelines have been published in final form [12]; our synthesis is, therefore, a timely commentary on a finalized recommendation rather than on a provisional document. Our results are compatible with the direction of that recommendation for undifferentiated sepsis, in which no robust effect was found, but they identify septic shock as the setting in which the case-by-case exception is most defensible: the estimate was larger, internally consistent across trials, and independent of the variance estimator, whereas a uniform preference for crystalloids alone would not be supported by the present data. Read alongside the acknowledgement that the mortality evidence remains of low certainty [13,14], this pattern is consistent with a benefit that is real but modest and concentrated in the most severe patients, and our analysis supplies the direction and approximate magnitude of that benefit while remaining candid about its imprecision.

### Limitations

However, several limitations temper these conclusions. First, the certainty of evidence is low, driven by imprecision; the confidence interval of the overall estimate remains compatible with no effect and indirectness arising from the contribution of extracted strata and mixed-severity populations. Second, the septic-shock analysis relies in part on subgroup data, including the post hoc septic-shock stratum of ALBIOS [17], which was not the trial’s pre-specified primary comparison; subgroup estimates are inherently more fragile than trial-level primary outcomes. The credibility of the septic-shock effect modification was formally rated as low using the ICEMAN instrument (Supplementary File S6), concordant with the low GRADE certainty. Third, several contributing trials were small and raised concerns regarding risk of bias and non-blinded clinicians. In the study by Dong et al. [35], the albumin concentration was not reported and could not be confirmed with the corresponding author, and the pre-registered exclusion of that trial did not materially change the pooled estimate (RR 0.94). The inclusion of one trial available only as a congress abstract (Goyal & Agarwal [38]) introduces a risk of incomplete reporting, reflected in its “some concerns” risk-of-bias rating; the results were robust to its removal in the leave-one-out analysis. Fourth, the main septic-shock analysis incorporates the refractory-hypotension stratum of ICARUS-ED [28]. This subgroup was pre-specified in the trial’s analysis plan but reported as exploratory, and it was derived from a single-center pilot feasibility trial whose primary outcome was systolic blood pressure at 24 h. Eligibility for the stratum rested on hypotension refractory to a 1000 mL fluid bolus rather than on vasopressor-dependent septic shock, and mortality, although added as an endpoint before enrolment commenced, was not listed in the trial registry. Strata of this kind remain less robust than trial-level comparisons; the mortality counts were verified against the trial’s published online supplement, and the estimate restricted to the five contributions in which the septic shock population was defined by shock criteria at enrolment was essentially identical (RR 0.90), so this stratum does not drive the result. Fifth, with eight trials, the formal assessment of publication bias was underpowered. Additional limitations concern the review process itself. The search for the period before 21 March 2024 was adopted from Bai et al. rather than re-run independently, so the baseline evidence depends on their eligibility and search decisions; primary screening was performed by a single reviewer with partial co-screening; the primary outcome pooled mortality across heterogeneous time points (in-hospital, 28-, 30-, and 90-day); and the analysis departed from the registered protocol in the respects detailed in Section 2.9, including a variance-estimation strategy that was only partly pre-planned. In addition, trials of undifferentiated shock that might contain an extractable septic-shock stratum were not specifically sought, and one substantially weighted contributing trial (EARSS, 12.1% of the primary analysis) is available only as a congress abstract; congress abstracts were eligible under the pre-registered protocol, and the result was robust to leave-one-out omission. Finally, one author (C.J.W.) has received fees from albumin manufacturers, as declared in the Conflicts of Interest statement.

The completed but unpublished ALBIOSS-BALANCED trial (NCT03654001) [27], a large septic-shock RCT of 20% albumin, will materially inform this estimate once published and may either sharpen or attenuate the septic-shock signal; we have registered our intention to incorporate it in an update after publication. This review was restricted to all-cause mortality; safety and secondary clinical outcomes (renal outcomes, pulmonary edema, treatment-related adverse events, and fluid balance) were not extracted and remain to be synthesized in future work.

## 5. Conclusions

In adults with sepsis, hyperoncotic albumin (20%) did not robustly reduce all-cause mortality when compared with crystalloids, and the apparent overall significance obtained under one variance estimator did not survive a transparent sensitivity analysis. A concentrated, internally consistent, and biologically plausible signal in septic shock—a ~10% relative risk reduction convergent with earlier syntheses and consistent with the individually inconclusive ARISS trial [18]—is consistent with the case-by-case role for albumin in septic shock that the 2026 Surviving Sepsis Campaign guidelines permit, rather than a uniform preference for crystalloids alone. This signal, however, derives from low-certainty evidence and partly post hoc subgroup strata and does not constitute a recommendation for routine use; the overall analysis does not support routine albumin across undifferentiated sepsis, and adequately powered trials are required to confirm the septic-shock effect. The forthcoming ALBIOSS-BALANCED trial is anticipated to refine the magnitude of this effect.

## Figures and Tables

**Table 1:** Characteristics of the included randomized controlled trials: design and population.

Study (Year)	Country; Centers	Design; Randomization	Sepsis Definition	Severity and Comorbidity	Sample Size (Albumin/Crystalloids)
EARSS—Charpentier & Mira (2011) [39]	France; 29 centers	open label; allocation concealment not reported; multi-center	clinical septic shock; vasopressor requirement within 6 h	Septic shock: 100% (by definition). Cirrhosis: excluded. SOFA: median 10 (IQR 8–12).	*n* = 798 (399/399)
ALBIOS—Caironi et al. (2014) [17]	Italy; 100 centers	open label; centralized computer-generated blinded randomization stratified by ICU and time to enrolment; multi-center	Sepsis-1 (ACCP/SCCM 1992, Bone); severe sepsis within 24 h of ICU admission	Septic shock: 62.7% (565/903 albumin; 570/907 crystalloids). Cirrhosis: excluded (liver disease, 1.4% vs. 1.5%). SOFA: median 8 (IQR 6–10).	*n* = 1810 analyzed (903/907); 1818 randomized
Kakaei et al. (2017) [40]	Iran; 1 center (Tabriz)	randomized pilot trial; allocation concealment not described; single center	SIRS-based (≥2 SIRS criteria + confirmed infection source + fluid requirement; no consensus definition cited)	Septic shock: not reported as a proportion; vasopressor use was not specified as an inclusion criterion. Cirrhosis was not specifically excluded. SOFA: median 6.1 (IQR 3.5) in the crystalloid arm, median 5.2 (IQR 1.6) in the albumin arm; *p* = 0.99.	*n* = 20 (10/10)
ALPS—Maiwall et al. (2022) [36]	India; 1 center (New Delhi, ILBS)	open label; computer-generated randomization; single center	MAP < 65 mmHg + suspected/confirmed infection (clinical)	Septic shock: 100% (according to the inclusion criteria). Cirrhosis: 100% (cirrhosis-specific cohort). SOFA: median 10 (IQR 8–12) albumin arm; median 10 (IQR 8–13) control arm.	*n* = 100 (50/50)
Dong et al. (2025) [35]	China; 1 center (Shijiazhuang, Hebei)	open label; 2:1 computer randomization; single center	Sepsis-3 (SSC 2021); fluid responsiveness assessed by CVP, PLR, mini-fluid challenge, echocardiography	Septic shock: 67% (according to published data). Cirrhosis: not reported. SOFA: mean 8.5 (SD 2.3) albumin; mean 8.2 (SD 2.1) control group.	*n* = 66 (44/22)
Goyal & Agarwal (2025) [38]	India; 1 center (Udaipur, Rajasthan)	open label; allocation concealment adequate (sequentially numbered sealed opaque envelopes); single center	sepsis-associated hypotension (MAP < 65 mmHg after ≥30 mL/kg crystalloids); SSC criteria	Septic shock: 100% (by inclusion criteria). Cirrhosis: 100% (cirrhosis-specific cohort). SOFA: not reported.	*n* = 60 (30/30)
ICARUS-ED—Williams et al. (2025) [28]	Australia; 1 center (Brisbane)	open label; computer randomization; single center; no prior fluid loading required for eligibility	suspected infection + hypoperfusion (SBP < 90 mmHg or lactate ≥ 4.0 mmol/L)	Mixed sepsis/hypoperfusion cohort; not restricted to patients with septic shock. Refractory hypotension (SBP < 90 mmHg after ≥1000 mL fluid) was used for the septic-shock subgroup in 386/463 (83%) patients. Cirrhosis: excluded. SOFA: median 4 (IQR 2–7) in both arms.	*n* = 463 analyzed (232/231); 464 randomized
ARISS—Sakr et al. (2026) [18]	Germany; 23 centers	open label; computer randomization; multi-center; terminated early	Sepsis-3 [1]; vasopressor requirement ≥ 1 h despite adequate fluid resuscitation; lactate < 18 mg/dL	Septic shock: 100% (by definition). Cirrhosis: not reported. SOFA: median 11 (IQR 8–13) albumin; median 11 (IQR 9–14) control.	*n* = 440 (222/218)

ACCP, American College of Chest Physicians; CVP, central venous pressure; ICU, intensive care unit; ILBS, Institute of Liver and Biliary Sciences; IQR, interquartile range; MAP, mean arterial pressure; PLR, passive leg raising; SBP, systolic blood pressure; SCCM, Society of Critical Care Medicine; SD, standard deviation; SIRS, systemic inflammatory response syndrome; SOFA, Sequential Organ Failure Assessment; SSC, Surviving Sepsis Campaign.

**Table 2:** Interventions, comparators, and mortality outcomes of the included trials.

Study (Year)	Albumin Intervention (Concentration; Dose; Trigger; Duration)	Comparator	Primary Endpoint	Mortality Data (Events/*n*, Albumin vs. Crystalloid; Effect [95% CI]; *p*)
EARSS—Charpentier & Mira (2011) [39]	Concentration: 20% (LFB). Dose: 100 mL q8h. Trigger: vasopressor-dependent septic shock ≤ 6 h. Duration: 3 days.	0.9% NaCl; 100 mL q8h × 3 days.	28-day all-cause mortality	28 d: 96/399 (24.1%) vs. 105/399 (26.3%); RR not reported; *p* = NS; no CI.
ALBIOS—Caironi et al. (2014) [17]	Concentration: 20%. Dose: 300 mL loading, then daily to target serum albumin ≥ 30 g/L. Trigger: severe sepsis at ICU admission. Duration: max. 28 days or ICU discharge.	Crystalloids alone (no synthetic colloids; type at the clinician’s discretion).	28-day all-cause mortality	28 d: 285/895 (31.8%) vs. 288/900 (32.0%); RR 1.00 (95% CI 0.87–1.14); *p* = 0.94. 90 d: 365/888 (41.1%) vs. 389/893 (43.6%); RR 0.94 (95% CI 0.85–1.05); *p* = 0.29. Post hoc septic-shock subgroup: 90 d RR 0.87 (95% CI 0.77–0.99).
Kakaei et al. (2017) [40]	Concentration: 20%. Dose: Two vials added to every 1 L of crystalloids. Trigger: severe sepsis with fluid requirement. Duration of treatment was not specified.	Crystalloids alone (type not specified).	28-day and 90-day all-cause mortality (co-primary)	28 d: 1/10 (10%) vs. 3/10 (30%); *p* = 0.58; no CI. 90 d: 4/10 (40%) vs. 5/10 (50%); *p* = 0.50; no CI.
ALPS—Maiwall et al. (2022) [36]	Concentration: 20%. Dose: 0.5–1.0 g/kg over 3 h, continued for 24 h. Trigger: septic shock in cirrhosis. Duration: 24 h.	Plasmalyte: 30 mL/kg over 3 h, continued for 24 h.	MAP > 65 mmHg at 3 h without vasopressors (hemodynamic)	28 d (secondary): 29/50 (58%) vs. 31/50 (62%); log-rank *p* = 0.57; RR not reported; no CI.
Dong et al. (2025) [35]	Concentration: 20% [not stated in publication; 20% assumed; author inquiry unanswered, March 2026]. Dose: 20 g intravenous bolus, followed by balanced crystalloids. Trigger: fluid-responsive sepsis/septic shock. Duration: single bolus, repeated if fluid responsive.	Balanced crystalloid: 30 mL/kg; hourly volume reassessment.	plasma syndecan-1 concentration over time (glycocalyx biomarker; surrogate endpoint)	28 d (secondary): 9/44 (20.5%) vs. 3/22 (13.6%); *p* = 0.498; no CI. 90 d (secondary): 15/44 (34.1%) vs. 6/22 (27.3%); *p* = 0.575; no CI.
Goyal & Agarwal (2025) [38]	Concentration: 20%. Dose: 1.5 g/kg over 3 h (day 1); optional 2nd dose 1.0 g/kg on day 3. Trigger: septic shock in cirrhosis. Duration: up to two doses over 3 days.	Plasmalyte: 30 mL/kg over 3 h.	reversal of hypotension (MAP ≥ 65 mmHg within 6 h without vasopressors; hemodynamic)	In-hospital (secondary): 6/30 (20%) vs. 8/30 (26.7%); *p* = 0.54; OR 0.69; no CI.
ICARUS-ED—Williams et al. (2025) [28]	Concentration: 20% (Alburex 20; CSL Behring). Dose: 400 mL (fixed 80 g bolus) over 4 h. Trigger: sepsis-associated hypoperfusion at ED presentation. Duration: single fixed bolus.	Standard crystalloids (0.9% saline and Hartmann’s); protocol specified no albumin (minimal control-arm crossover: mean ~16 mL 20% + ~146 mL 4% albumin for 72 h).	systolic blood pressure at 24 h (hemodynamic)	30 d (secondary): 38/232 (16.4%) vs. 36/231 (15.6%); *p* = NS; RR not reported.
ARISS—Sakr et al. (2026) [18]	Concentration: 20%. Dose: 60 g loading over 2–3 h; maintenance 40–80 g/day to achieve a target serum albumin level of ≥3.0 g/dL (30 g/L). Trigger: septic shock ≤ 24 h. Duration: max. 28 days (ICU stay).	Standard crystalloids (type at clinician discretion); albumin permitted at physician discretion (48.6% of the control arm received albumin).	90-day all-cause mortality	90 d: 91/210 (43.3%) vs. 96/209 (45.9%); RR 0.94 (95% CI 0.76–1.17); *p* = 0.71. 28 d: 66/213 (31.0%) vs. 80/210 (38.1%); *p* = NS; RR not reported.

CI, confidence interval; ED, emergency department; ICU, intensive care unit; i.v., intravenous; LFB, Laboratoire français de Fractionnement et de Biotechnologies; MAP, mean arterial pressure; NaCl, sodium chloride; NS, not significant; OR, odds ratio.

**Table 3:** Pooled risk ratio for the primary outcome under four variance estimation methods (identical point estimate; k = 8; I^2^ = 0%).

Method	RR	95% CI	*p*-Value
Standard random effects (z)	0.94	0.87–1.03	0.17
Raw HKSJ	0.94	0.91–0.98	0.01
Truncated HKSJ (factor ≥ 1)	0.94	0.85–1.04	0.21
Fixed-effects	0.94	0.87–1.03	0.17

RR, risk ratio; CI, confidence interval; HKSJ, Hartung–Knapp–Sidik–Jonkman.

**Table 4:** Sensitivity analyses for the primary outcome (all-cause mortality at the longest available follow-up).

Analysis	k	*n*	RR	95% CI (Standard)	95% CI (HKSJ)	I^2^
Primary analysis (all trials)	8	3707	0.94	0.87–1.03	0.91–0.98	0%
Restriction to ≥50 participants per arm	5	3561	0.94	0.87–1.03	0.91–0.98	0%
Restriction to large RCTs (≥400 total)	4	3461	0.94	0.86–1.03	0.90–0.99	0%
Exclusion of Dong et al. (combination design)	7	3641	0.94	0.86–1.02	0.91–0.97	0%
Exclusion of ALPS (cirrhosis specific)	7	3607	0.94	0.87–1.03	0.90–0.99	0%
Leave-one-out (range across 8 refits)	7	—	0.94–0.95	—	—	0%

The point estimates were invariant across all analyses. Standard intervals were obtained from the random-effects model with a normal (z) distribution. RR, risk ratio; CI, confidence interval; HKSJ, Hartung–Knapp–Sidik–Jonkman.

**Table 5:** GRADE Summary of Findings: Hyperoncotic albumin (20%) versus crystalloids in adults with sepsis.

Outcome (Follow-Up)	No. Participants (Studies)	Relative Effect, RR (95% CI)	Assumed Risk with Crystalloids (Per 1000)	Risk Difference with Albumin (Per 1000; 95% CI)	Certainty (GRADE)
All-cause mortality, longest follow-up	3707 (8 RCTs)	0.94 (0.87–1.03)	367	21 fewer (48 fewer to 9 more)	⊕⊕○○ Low ^a,b^
90-day mortality	2286 (4 RCTs)	0.95 (0.86–1.04)	437	24 fewer (61 fewer to 18 more)	⊕⊕○○ Low ^a,b^
28-day mortality	3202 (6 RCTs)	0.95 (0.85–1.05)	321	17 fewer (47 fewer to 15 more)	⊕⊕○○ Low ^a,b^
All-cause mortality in septic shock (subgroup)	2884 (6 RCTs)	0.90 (0.82–0.99)	383	37 fewer (67 fewer to 3 fewer)	⊕⊕○○ Low ^a,c^

RR: risk ratio; CI: confidence interval. Certainty starts at a high level (randomized trials) and is downgraded as follows: ^a^ imprecision—the 95% confidence interval is compatible with a clinically relevant benefit, no effect, and potential harm (in the case of septic shock, the interval remains compatible with no effect); ^b^ indirectness—several trials were not primarily designed or powered for mortality, and the analysis incorporated mixed-severity populations; and ^c^ indirectness—the subgroup analysis used partly post hoc and separately extracted strata (ALBIOS [17] and ICARUS-ED [28]). The relative effects are from the standard random-effects model, and the variance-method dependence of the primary interval is shown in Table 3. Symbols: ⊕⊕○○ = low certainty.

## Data Availability

The original contributions presented in this study are included in the article/Supplementary Materials; no new data were generated. Further inquiries can be directed to the corresponding author.

## Supplementary Materials

The following supporting information can be downloaded at https://www.mdpi.com/article/10.3390/medsci14050550/s1, Supplementary File S1: Search strategy, search terms, and records retrieved; Supplementary File S2: Risk-of-bias assessment (RoB 2): domain-level judgements for the primary outcome (all-cause mortality at the longest available follow-up); Supplementary File S3: Records assessed at the full-text eligibility stage (*n* = 14); Supplementary File S4: Completed PRISMA 2020 checklist; Supplementary File S5: Dataset: extracted all-cause mortality events and denominators; Supplementary File S6: Credibility of the septic-shock subgroup effect (ICEMAN assessment). References [25,47] are cited in the supplementary materials.

## Abbreviations

The following abbreviations are used in this manuscript:

ABC	Albumin versus Balanced Crystalloid
ACCP	American College of Chest Physicians
ALBIOS	Albumin Italian Outcome Sepsis
ALBIOSS	Albumin Italian Outcome Septic Shock
ALBUS	Albumin Use in Surgical Septic Shock
ALPS	Albumin versus Crystalloid in Cirrhotics with Septic Shock
ARISS	Albumin Replacement Therapy in Septic Shock
CI	Confidence interval
CVP	Central venous pressure
EARSS	Early Albumin Resuscitation during Septic Shock
ED	Emergency department
ESICM	European Society of Intensive Care Medicine
GRADE	Grading of Recommendations Assessment, Development and Evaluation
HKSJ	Hartung–Knapp–Sidik–Jonkman
ICARUS-ED	Intervention with Concentrated Albumin for Undifferentiated Sepsis in the Emergency Department
ICU	Intensive care unit
ILBS	Institute of Liver and Biliary Sciences
IQR	Interquartile range
i.v.	Intravenous
LFB	Laboratoire français de Fractionnement et de Biotechnologies
MAP	Mean arterial pressure
NaCl	Sodium chloride
NS	Not significant
OR	Odds ratio
PICOS	Participants, Intervention, Comparator, Outcomes, Study design
PLR	Passive leg raising
PRISMA	Preferred Reporting Items for Systematic Reviews and Meta-Analyses
RCT	Randomized controlled trial
REML	Restricted maximum likelihood
RM	Random effects model
RoB	Risk of bias
RR	Relative risk
SBP	Systolic blood pressure
SCCM	Society of Critical Care Medicine
SD	Standard deviation
SIRS	Systemic inflammatory response syndrome
SOFA	Sequential Organ Failure Assessment
SSC	Surviving Sepsis Campaign
WHO	World Health Organization
