# Supplementary material for: Hyperoncotic Albumin (20%) Versus Crystalloids for Mortality in Adults with Sepsis and Septic Shock: An Updated Systematic Review and Meta-Analysis
Source: Med Sci (Basel). 2026 Sep 7;14(5):550. doi: 10.3390/medsci14050550 (PMC13609289; doi:10.3390/medsci14050550)
Supplement: Supplementary file 1 [file medsci-14-00550-s001.zip › SupplFile_S1_SearchStrategy-done_R1.pdf]

medical sciences

MDPI

Supplementary File S1. Search strategy, search terms, and records retrieved.

The update search was conducted on 10 March 2026 and covered the period from 21 March 2024 — the end of the search by Bai et al. (2024), whose strategy was adopted for the preceding period — to the search date. Three databases were searched: MEDLINE (via PubMed), EMBASE, and the Cochrane Central Register of Controlled Trials (CENTRAL). No language restriction was applied. One additional trial (ALBIOSS-BALANCED, NCT03654001) was identified separately through the ClinicalTrials.gov register. The search strings are reproduced verbatim in Table S1.1, and the yield of records is summarised in Table S1.2.

Table S1.1. Database search strings.

Database	Search period	Search string
MEDLINE (PubMed)	21 March 2024 – 10 March 2026	`((sepsis [All Fields]) OR (septic shock [All Fields])) AND ((albumin [All Fields]) OR (colloid [All Fields])) AND ((randomized [All Fields]) OR (randomised [All Fields]) OR (clinical trial [All Fields]))`
EMBASE	2024–2026 ([/py] year filter)	`('sepsis'/exp OR sepsis OR 'septic shock'/exp OR 'septic shock' OR (septic AND ('shock'/exp OR shock)) OR 'severe sepsis'/exp OR 'severe sepsis' OR 'infection'/exp OR infection OR 'organ failure'/exp OR 'organ failure' OR 'multi-organ failure' OR 'multi-organ dysfunction syndrome'/exp OR 'organ dysfunction'/exp OR 'organ dysfunction') AND ('20% albumin' OR (20% AND ('albumin'/exp OR albumin)) OR '25% albumin' OR (25% AND ('albumin'/exp OR albumin)) OR 'human albumin solution' OR 'hyperoncotic resuscitation fluid' OR 'intravenous fluid'/exp OR 'albumin supplement' OR 'albumin supplementation') AND ('crystalloid'/exp OR 'crystalloid') AND ('mortality'/exp OR 'mortality') AND [2024-2026]/py`
Cochrane CENTRAL	1 March 2024 – 10 March 2026	`(sepsis OR "septic shock") AND (albumin OR "human albumin") AND (crystalloid OR saline OR "normal saline" OR "Hartmann's" OR "Plasmalyte") AND (randomized OR randomised OR "clinical trial")`

Emtree terms in the EMBASE strategy were exploded (/exp). Search strings are reproduced exactly as executed.

Table S1.2. Records retrieved and reconciliation with the PRISMA 2020 flow diagram.

Source	Total hits	After date filter	After deduplication
MEDLINE (PubMed)	1039	128	
EMBASE	419	64	
Cochrane CENTRAL	112	19	
**Databases, subtotal**	**1570**	**211**	**198**
ClinicalTrials.gov register	—	1	1
**Total (records screened, Figure 1)**	**—**	**212**	**199**

Deduplication (13 duplicate records removed) was performed across the pooled results of the three databases (211 → 198). The register record (ALBIOSS-BALANCED, NCT03654001) was identified separately and did not form part of the database yield. The 198 de-duplicated database records together with this single register record constitute the 199 records screened in the PRISMA 2020 flow diagram (Figure 1). *Em dash (—) denotes not applicable.*
