# Supplementary material for: Hyperoncotic Albumin (20%) Versus Crystalloids for Mortality in Adults with Sepsis and Septic Shock: An Updated Systematic Review and Meta-Analysis
Source: Med Sci (Basel). 2026 Sep 7;14(5):550. doi: 10.3390/medsci14050550 (PMC13609289; doi:10.3390/medsci14050550)
Supplement: Supplementary file 1 [file medsci-14-00550-s001.zip › SupplFile_S2_RiskOfBias-done_R1.pdf]

medical sciences

MDPI

**Supplementary File S2.** Risk-of-bias assessment (RoB 2): domain-level judgements for the primary outcome (all-cause mortality at the longest available follow-up).

Risk of bias was assessed with the Cochrane Risk of Bias tool for randomised trials, version 2 (RoB 2), for each included study. Judgements for each domain and the overall judgement are shown below; the same assessment is displayed graphically in Figure 2.

**Table S2.** Risk-of-bias assessment (RoB 2): domain-level judgements for the primary outcome (all-cause mortality at the longest available follow-up).

| Study | D1 | D2 | D3 | D4 | D5 | Overall |
|---|---|---|---|---|---|---|
| EARSS (Charpentier & Mira, 2011) | Some concerns | Some concerns | High | Low | High | High |
| ALBIOS (Caironi et al., 2014) | Low | Low | Low | Low | Low | Low |
| Kakaei et al. (2017) | Some concerns | Some concerns | Low | Low | Some concerns | High |
| ALPS (Maiwall et al., 2022) | Low | Some concerns | Low | Low | Low | Some concerns |
| Dong et al. (2025) | Some concerns | Some concerns | Some concerns | Some concerns | High | High |
| Goyal & Agarwal (2025) | Low | Some concerns | Some concerns | Some concerns | High | High |
| ICARUS-ED (Williams et al., 2025) | Low | Low | Low | Low | Some concerns | Some concerns |
| ARISS (Sakr et al., 2026) | Low | Some concerns | Low | Low | Low | Some concerns |

**Domains.** D1, bias arising from the randomisation process; D2, bias due to deviations from the intended interventions; D3, bias due to missing outcome data; D4, bias in measurement of the outcome; D5, bias in selection of the reported result.

**Judgements.** Each domain and the overall risk of bias were rated as Low, Some concerns, or High. The overall judgement is the least favourable domain-level judgement, in accordance with the RoB 2 algorithm.

**Assessment.** Risk of bias was rated for the primary outcome (all-cause mortality at the longest available follow-up) by one reviewer (C.J.W.) and verified by a second (G.T.); disagreements were resolved by discussion. None of the trials blinded participants or clinicians, given the impracticability of blinding fluid interventions; the objective mortality outcome was nonetheless rated at low risk for measurement of the outcome (D4).
