# Supplementary material for: Hyperoncotic Albumin (20%) Versus Crystalloids for Mortality in Adults with Sepsis and Septic Shock: An Updated Systematic Review and Meta-Analysis
Source: Med Sci (Basel). 2026 Sep 7;14(5):550. doi: 10.3390/medsci14050550 (PMC13609289; doi:10.3390/medsci14050550)
Supplement: Supplementary file 1 [file medsci-14-00550-s001.zip › SupplFile_S3_FullTextRecords-done_R1.pdf]

medical sciences

MDPI

Supplementary File S3. Records assessed at the full-text eligibility stage (*n* = 14).

Records are listed alphabetically by first author. Decisions and exclusion reasons follow the pre-specified PICOS eligibility criteria (Supplementary File S1) and correspond to the PRISMA 2020 flow diagram (Figure 1). Included (*n* = 4), excluded (*n* = 10). Standardized exclusion reasons: intervention not hyperoncotic albumin (5% or no albumin); comparator not crystalloid-only; outcome without a time-defined mortality endpoint; ongoing trial without data; completed trial without results; record outside the update-search window (before 21 March 2024); and companion or duplicate records of studies already listed (including the trial registration of the included ICARUS-ED study). One further duplicate record (a second index of the J. Crit. Care report of the MICALB21 trial) was removed at the deduplication stage and is therefore not listed here.

Table S3. Records assessed at the full-text eligibility stage (*n* = 14).

Number	Full citation	Decision	Reason / note
1	Caironi, P.; et al. ALBumin Italian Outcome Septic Shock–BALANCED (ALBIOSS-BALANCED). ClinicalTrials.gov Identifier NCT03654001; completed, unpublished, 2026.	Excluded	Completed trial without results (register entry; unpublished, no extractable outcome data).
2	Cusack, R.; et al. Albumin Enhances Microvascular Reactivity in Sepsis: Insights from Near-Infrared Spectroscopy and Vascular Occlusion Testing. *J. Clin. Med.* 2025, 14, 4982. https://doi.org/10.3390/jcm14144982.	Excluded	Outcome — microvascular/near-infrared-spectroscopy surrogate endpoint; no time-defined mortality data.
3	Cusack, R.A.F.; Rodriguez, A.; Cantan, B.; et al. Microcirculation properties of 20% albumin in sepsis; a randomised controlled trial. *J. Crit. Care* 2025, 87, 155039. https://doi.org/10.1016/j.jcrc.2025.155039.	Excluded	Companion report of the MICALB21 trial (secondary report of an already-excluded study).
4	Dong, L.; et al. [Impact and clinical significance of different types of fluid resuscitation on the glycocalyx in patients with sepsis]. *Zhonghua Wei Zhong Bing Ji Jiu Yi Xue* 2025, 37, 237–244. https://doi.org/10.3760/cma.j.cn121430-20240711-00583.	Included	Meets all eligibility criteria: hyperoncotic (20%) albumin versus balanced crystalloid; sepsis; all-cause mortality reported.
5	Goyal, C.; Agarwal, D. Efficacy and Safety of 20% Albumin Compared to Plasmalyte in Reversing Sepsis-Related Hypotension in Cirrhotic Patients. *SSR Inst. Int. J. Life Sci.* 2025, 11, 8340–8347.	Included	Meets all eligibility criteria: 20% albumin versus Plasma-Lyte; sepsis-related hypotension; in-hospital mortality reported.
6	Gray, A.J.; et al. Albumin Versus Balanced Crystalloid for the Early Resuscitation of Sepsis: An Open Parallel-Group Randomized Feasibility Trial (ABC-Sepsis). *Crit. Care Med.* 2024, 52, 1520–1532. https://doi.org/10.1097/CCM.0000000000006348.	Excluded	Intervention — iso-oncotic 5% human albumin solution (not hyperoncotic ≥20%).
7	Gupta, T.; et al. Comparative Study of Terlipressin and Noradrenaline as Vasopressors in Patients With Acute-on-chronic Liver Failure. *J. Clin. Exp. Hepatol.* 2025, 15, 102494. https://doi.org/10.1016/j.jceh.2024.102494.	Excluded	Intervention — vasopressor trial (terlipressin versus noradrenaline); no albumin.
8	Human Albumin for Resuscitation in Surgical Septic Shock: a Randomized Controlled Trial (ALBUS Study). ClinicalTrials.gov Identifier NCT07212582; Mahidol University, 2025.	Excluded	Ongoing trial (not yet recruiting; 20% albumin versus standard care; estimated completion 2030); no outcome data.
9	Jain, A.; Tewethia, H.V.; Rajan, V.; et al. Efficacy of 5% albumin vs plasmalyte in combination with 20% albumin for	Excluded	Companion report of the TOP-204 trial (Maiwall et al.); secondary

	fluid resuscitation in cirrhosis with sepsis. *J. Clin. Exp. Hepatol.* 2025, 15 (abstract).		report of an already-excluded study.
10	Maiwall, R.; Jain, A.; Rajan, V.; et al. Efficacy of 5% albumin versus plasmalyte in combination with 20% albumin for fluid resuscitation in cirrhosis with sepsis (TOP-204). *J. Hepatol.* 2025, 82 (Suppl.), S131–S132 (abstract).	Excluded	Comparator — 5% albumin plus 20% albumin combination versus Plasma-Lyte (not crystalloid-only).
11	Sakr, Y.; et al. Albumin Replacement Therapy in Septic Shock: A Randomized Clinical Trial (ARISS). *JAMA Netw. Open* 2026, 9, e2559297. https://doi.org/10.1001/jamanetworkopen.2025.59297.	Included	Meets all eligibility criteria: 20% albumin versus crystalloid; septic shock; 90-day and 28-day mortality reported.
12	Verma, A.; Maiwall, R.; Sahney, A.; Mitra, L.; Sarin, S.K. Comparison of 20% albumin versus crystalloid plasmalyte for fluid resuscitation in cirrhotics with sepsis-induced hypotension. *J. Hepatol.* 2021, 75 (Suppl.), S205–S293 (abstract).	Excluded	Record outside the update-search window (2021 conference abstract; before 21 March 2024).
13	Williams, J.M.; et al. Intervention With Concentrated Albumin for Undifferentiated Sepsis in the Emergency Department (ICARUS-ED): A Randomized Clinical Trial. *Ann. Emerg. Med.* 2025, 86, 59–69. https://doi.org/10.1016/j.annemergmed.2024.12.016.	Included	Meets all eligibility criteria: 20% albumin versus balanced crystalloid; undifferentiated sepsis (ED); all-cause mortality reported.
14	Williams, J.M.; et al. Intervention with Concentrated Albumin for Resuscitation of Undifferentiated Sepsis (ICARUS-ED) — trial registration (ANZCTR). 2024.	Excluded	Trial registration of the included ICARUS-ED study (duplicate record of report no. 13); consolidated.
