# Supplementary material for: Hyperoncotic Albumin (20%) Versus Crystalloids for Mortality in Adults with Sepsis and Septic Shock: An Updated Systematic Review and Meta-Analysis
Source: Med Sci (Basel). 2026 Sep 7;14(5):550. doi: 10.3390/medsci14050550 (PMC13609289; doi:10.3390/medsci14050550)
Supplement: Supplementary file 1 [file medsci-14-00550-s001.zip › SupplFile_S5_Dataset-done_R1.pdf]

medical sciences

MDPI

Supplementary File S5. Dataset: extracted all-cause mortality events and denominators.

This file provides the complete data underlying every analysis, to permit independent replication. The trial-level dataset (Table S5.1) is provided in machine-readable form as the accompanying comma-separated file (Supplementary File S5, .csv) and reproduces the primary, secondary, trial-level subgroup (cirrhosis; exclusively septic-shock trials), and sensitivity analyses when processed with the R/metafor script described in the Methods. The septic-shock subgroup reported in the main text (k = 6) additionally incorporates the separately extractable ALBIOS and ICARUS-ED strata documented in Table S5.2.

Table S5. 1. Trial-level all-cause mortality data (analysis input).

Study	Time point	Albumin (deaths/n)	Crystalloid (deaths/n)	Primary	Trial-level classification
EARSS	28 d	96/399	105/399	Yes	Exclusively septic shock; large RCT
ALBIOS	28 d	285/895	288/900	No	Large RCT
ALBIOS	90 d	365/888	389/893	Yes	Large RCT
Kakaei	28 d	1/10	3/10	No	Severe sepsis without shock
Kakaei	90 d	4/10	5/10	Yes	Severe sepsis without shock
Dong	28 d	9/44	3/22	No	Mixed sepsis/septic shock
Dong	90 d	15/44	6/22	Yes	Mixed sepsis/septic shock
ALPS	28 d	29/50	31/50	Yes	Exclusively septic shock; cirrhosis
Goyal & Agarwal	In-hospital	6/30	8/30	Yes	Exclusively septic shock; cirrhosis
ICARUS-ED	30 d	38/232	36/231	Yes	Mixed sepsis/septic shock; large RCT
ARISS	28 d	66/213	80/210	No	Exclusively septic shock; large RCT; early-terminated
ARISS	90 d	91/210	96/209	Yes	Exclusively septic shock; large RCT; early-terminated

Codebook (column names as used in the .csv file: study = trial; timepoint = mortality time point; ai, n1i = deaths and total in the albumin arm; ci, n2i = deaths and total in the crystalloid arm; longest = 1 marks the row used in the primary analysis (longest available follow-up); shock = 1 denotes a trial enrolling exclusively patients with septic shock; cirrhosis = 1 denotes a cirrhosis/acute-on-chronic-liver-failure population; large = 1 denotes a large RCT (≥ 400 total participants), used in the large-RCT sensitivity analysis; early = 1 denotes an early-terminated trial. Deaths/n are shown in Table S5.1 as deaths/total.

Table S5. 2. Composition of the severity subgroups and provenance of the extracted strata.

Contribution	Albumin (deaths/n)	Crystalloid (deaths/n)	Source / note
**Septic-shock subgroup (k = 6; n = 2,884)**			

Contribution	Albumin (deaths/n)	Crystalloid (deaths/n)	Source / note
EARSS (28 d)	96/399	105/399	Exclusively septic-shock trial (Table S5.1).
ALPS (28 d)	29/50	31/50	Exclusively septic-shock trial (Table S5.1).
Goyal & Agarwal (in-hospital)	6/30	8/30	Exclusively septic-shock trial (Table S5.1).
ARISS (90 d)	91/210	96/209	Exclusively septic-shock trial (Table S5.1).
ALBIOS, septic-shock stratum (90 d)	243/558	281/563	Post-hoc septic-shock subgroup (n = 1,121); RR 0.87 (95% CI 0.77–0.99) (Caironi et al. 2014).
ICARUS-ED, refractory-hypotension stratum (30 d)	35/197	30/189	Extracted from Supplementary Table 6 of Williams et al. 2025.
**Sepsis-without-shock comparator subgroup**			
ALBIOS, non-shock stratum (90 d)	122/330	108/330	Non-shock stratum (n = 660); RR 1.13 (95% CI 0.92–1.39). Obtained as the whole-trial 90-day mortality minus the septic-shock stratum (Caironi et al. 2014).
ICARUS-ED, non-shock complement (30 d)	3/35	6/42	Whole trial (Table S5.1) minus the refractory-hypotension stratum.
Kakaei (90 d)	4/10	5/10	Severe sepsis without shock (Table S5.1).

The ALBIOS strata correspond to the 90-day mortality subgroups (septic shock, n = 1,121; sepsis without shock, n = 660); the non-shock counts and the ICARUS-ED non-shock complement were obtained by subtracting the corresponding shock stratum from the whole-trial mortality counts.
