# Supplementary material for: Hyperoncotic Albumin (20%) Versus Crystalloids for Mortality in Adults with Sepsis and Septic Shock: An Updated Systematic Review and Meta-Analysis
Source: Med Sci (Basel). 2026 Sep 7;14(5):550. doi: 10.3390/medsci14050550 (PMC13609289; doi:10.3390/medsci14050550)
Supplement: Supplementary file 1 [file medsci-14-00550-s001.zip › SupplFile_S6_ICEMAN-done_R1.pdf]

medical sciences

MDPI

Supplementary File S6. Credibility of the septic-shock subgroup effect (ICEMAN assessment).

The credibility of the effect modification of all-cause mortality by disease severity (septic shock versus sepsis without shock) was assessed using the Instrument to assess the Credibility of Effect Modification Analyses (ICEMAN) for meta-analyses. The assessment is summarized in Table S6.

Table S6. ICEMAN credibility assessment of the septic-shock subgroup effect.

ICEMAN criterion	Rating	Rationale for the septic-shock subgroup
Is the effect modifier (disease severity) a characteristic measured at baseline, before randomization?	Yes	Septic shock versus no shock was defined at enrolment, before treatment allocation.
Was the direction of the effect modification hypothesized a priori and supported by prior evidence?	Probably yes	The severity subgroup and the direction (greater benefit in shock) were pre-specified in the PROSPERO protocol on a biological rationale and are supported by the ALBIOS post-hoc subgroup and by prior syntheses (Bai et al.; Mendes et al.). At the trial level, however, the ALBIOS severity subgroup was itself post-hoc.
Does a formal test of interaction make chance an unlikely explanation?	No	The test for subgroup interaction was not statistically significant ($p = 0.08$).
Was only a small number of effect modifiers tested (limiting the risk of a spurious finding)?	Yes	Only two subgroups (severity and cirrhosis) were pre-specified; the analysis was not data-driven.
Was the effect modification based on within-study rather than between-study (ecological) comparisons?	Probably no	The subgroup rests substantially on between-study comparisons and on separately extracted strata (ALBIOS, ICARUS-ED); randomized within-study contrasts of shock versus no-shock were available for only part of the evidence. Between-study effect modification is more susceptible to confounding.
Is the effect modification consistent across studies and analytical choices?	Probably yes	The septic-shock point estimate was stable (RR 0.89–0.91) across the $k = 6$, $k = 5$, and $k = 3$ gradations, although these share the same dominant contributions.
**Overall ICEMAN credibility rating**	**Low**	**The credibility of the septic-shock effect modification is low, concordant with the low GRADE certainty of evidence, and supports a cautious, non-definitive interpretation.**

ICEMAN reference: Schandelmaier S, Briel M, Varadhan R, et al. Development of an instrument to assess the credibility of effect modification analyses (ICEMAN) in randomized controlled trials and meta-analyses. CMAJ 2020;192:E901–E906. doi:10.1503/cmaj.200077.
